# Task Complexity Modulates Sleep-Related Offline Learning in Sequential Motor Skills

**DOI:** 10.3389/fnhum.2017.00374

**Published:** 2017-07-25

**Authors:** Klaus Blischke, Andreas Malangré

**Affiliations:** Laboratory of Training Science, Department of Sport Science, Training Science, Saarland University Saarbrüecken, Germany

**Keywords:** sleep, memory consolidation, offline learning, task complexity, gross motor task, motor sequence learning

## Abstract

Recently, a number of authors have advocated the introduction of gross motor tasks into research on sleep-related motor offline learning. Such tasks are often designed to be more complex than traditional key-pressing tasks. However, until now, little effort has been undertaken to scrutinize the role of task complexity in any systematic way. Therefore, the effect of task complexity on the consolidation of gross motor sequence memory was examined by our group in a series of three experiments. Criterion tasks always required participants to produce unrestrained arm movement sequences by successively fitting a small peg into target holes on a pegboard. The sequences always followed a certain spatial pattern in the horizontal plane. The targets were visualized prior to each transport movement on a computer screen. The tasks differed with respect to sequence length and structural complexity. In each experiment, half of the participants initially learned the task in the morning and were retested 12 h later following a wake retention interval. The other half of the subjects underwent practice in the evening and was retested 12 h later following a night of sleep. The dependent variables were the error rate and total sequence execution time (inverse to the sequence execution speed). Performance generally improved during acquisition. The error rate was always low and remained stable during retention. The sequence execution time significantly decreased again following sleep but not after waking when the sequence length was long and structural complexity was high. However, sleep-related offline improvements were absent when the sequence length was short or when subjects performed a highly regular movement pattern. It is assumed that the occurrence of sleep-related offline performance improvements in sequential motor tasks is associated with a sufficient amount of motor task complexity.

## Introduction

For two decades, there has been mounting evidence showing that sleep plays a crucial role in learning and memory consolidation (Smith, 1995; Stickgold et al., 2001; Walker, 2005; Genzel et al., 2014). Drawing on the concept of an “active system consolidation” process (Born and Wilhelm, 2012), it is assumed that newly encoded skill representations are being actively (and repeatedly) reprocessed during sleep, the resulting in a qualitative reorganization and long-term stabilization of the respective memory representations. In this context, sleep has also been implicated in the consolidation process of *motor* skill memory following initial acquisition, with delayed learning being achieved in the absence of further practice. Specifically regarding motor *sequence* learning, significant offline improvements in speed and accuracy have repeatedly been found following nocturnal and diurnal sleep, whereas no such benefits were provided by equivalent wake periods (Walker et al., 2002; Fischer et al., 2005; Doyon et al., 2009; Malangré et al., 2014).

It should be noted, however, that the sleep-based enhancement hypothesis has recently been called into question. In several studies, researchers have identified a number of moderating variables that may account for at least a portion of the performance improvement following sleep (see Pan and Rickard, 2015, for a comprehensive review and meta-analysis). Some of these data even suggest that under some circumstances, skill performance after sleep may not be better than after a period of wakefulness (e.g., Backhaus et al., 2016), inviting a reconsideration of sleep’s theoretical role in the consolidation of procedural memories (Nettersheim et al., 2015). This debate is still unresolved at present.

As has been found repeatedly in motor sequence learning studies, sleep specifically enhances an allocentric spatial sequence representation, whereas an egocentric “motor” representation progressively develops with time (Cohen et al., 2005; Witt et al., 2010; Albouy et al., 2013). With regard to the neural substrates underlying the formation of these two components of sequence memory, recent fMRI-studies have shown that the abstract spatial map representing the schematic “gist” of motor sequence memory, is supposedly created through hippocampo-cortical activity during initial acquisition, while the motoric representation is assumed to be supported by the striato-cortical system. Antagonistic dynamic activity between the two systems during initial motor learning then is thought to condition subsequent sleep-related processes underlying enhanced sequence memory consolidation (Albouy et al., 2015).

Thus, it appears that sleep predominantly affords facilitation of the abstract, cognitive memory trace of a sequence, driven by early activity in the hippocampus and the frontal and parietal cortices. Development of this memory component is usually accompanied by at least rudimentary awareness of some of the regular features inherent to the sequence being learned. Therefore, this memory component is supposedly associated with declarative knowledge concerning the action’s goal as well as the type of sequence elements and their temporal order. Most likely, further declarative knowledge aspects of the task are liable to become part of this memory representation. This notion is also supported by the observation that subsequent sleep-dependent enhancement in performance has been consistently reported in *explicit* sequence learning conditions. In the respective paradigms, the sequence of elements to be performed is explicitly provided to the participants either prior to or throughout the initial practice (Albouy et al., 2013; Malangré et al., 2014). To the contrary, however, sleep appears to play no such critical role in implicit motor sequence learning (Robertson et al., 2004; Song et al., 2007; Nemeth et al., 2010).

Regarding explicitly acquired motor sequences, converging evidence from the behavioral sciences and neuroscience has uncovered some basic principles of sleep-dependent memory consolidation and continues to provide a deeper understanding of the underlying processes and mechanisms. At the same time, however, to date, surprisingly little attention has been paid to the question as to what extent these findings can be generalized across the domain of sequential motor tasks. Only recently have a number of studies deliberately incorporated *gross* motor tasks (Genzel et al., 2012; Kempler and Richmond, 2012; Morita et al., 2012; Al-Sharman and Siengsukon, 2013; Malangré et al., 2014; Gudberg et al., 2015; Hoedlmoser et al., 2015; Malangré and Blischke, 2016). Here, it has repeatedly been argued that nearly all the paradigms used previously to study sleep-dependent offline motor learning have been limited to relatively “simple” tasks (e.g., finger-to-thumb opposition tasks; serial reaction time tasks; sequential finger tapping tasks) compared to the motor tasks usually performed in daily life and thus might have only limited implications for applied areas such as vocational training, sports and rehabilitation. To overcome this limitation, in these recent studies, gross motor tasks involving unrestrained whole limb movements were introduced, including the upper and lower extremities.

Down the line, this choice of tasks was motivated by the respective authors’ objective to sufficiently increase the *task complexity* to adequately meet the functional requirements of real world applications. In this context, however, the concept of task complexity was usually conceived in a rather general way. For the most part and only vaguely defined, it has been associated with such diverse aspects such as, for example, sequence length, kinematic constraints, redundancy control, inter-limb coordination, the need to plan and execute goal-directed movements in Euclidian space, and reactions to different environmental (i.e., visual, auditory and proprioceptive) stimuli. However, so far as the extent to which these various aspects genuinely exercise influence over sleep-dependent motor memory consolidation processes remains open. One might even ask whether the notion of an all-encompassing concept of task complexity proves helpful at all with regard to elucidating the link between task demands and sleep-dependent memory consolidation in any sufficient detail.

This being said, surprisingly little effort has been undertaken to systematically examine the role of task complexity in sleep-dependent offline motor learning. To our knowledge, task complexity has been manipulated experimentally in only one study so far (Kuriyama et al., 2004). In this study, four groups of participants initially practiced uni- and bimanual key-pressing sequences of different lengths (five respective nine elements) and were retested 24 h later after a night of sleep. To produce the five-element configuration, the subjects had to press four numeric keys either with their left hand (unimanual group) or with two fingers from each hand (bimanual group). For the nine-element configuration, one group of subjects again pressed the four numeric keys with their left hands, while the corresponding bimanual group had to press eight numeric keys using four fingers from each hand. All groups significantly improved their sequence execution speed during initial training and then went on to significantly improve overnight. While the amount of offline improvement was similar for both of the short five-element sequences independently of number of hands involved, increasing the sequence length to nine elements in the unimanual condition resulted only in modest, but not significantly greater, offline improvements, whereas performing the nine-element bimanual task that used all eight digits produced dramatically greater overnight increases in execution speed compared to all the other groups. The authors attributed this significant result to the combined demand of memory load (i.e., sequence length) and the extent of movement coordination (i.e., the number of hands and digits required) and thus concluded that the more “complex” a task is, the larger the degree of sleep-dependent offline learning is.

Taking these propositions as a starting point to further elucidate the relationship between task complexity and motor memory consolidation, in our present work, we tried to (a) disentangle different complexity components, which are normally intertwined in many motor acts, and we also (b) adopted a gross motor skill for a criterion task that incorporated functional requirements typical of numerous activities of daily living. To this end we conducted a series of three experiments. In each experiment, the participants were to learn an unrestrained arm movement sequence of different levels of complexity. The task complexity was varied either with respect to *sequence length* (i.e., number of task elements) or with respect to *structural complexity* (defined by the amount of sequence regularity). Per definition, structural complexity increases as the regularity of the respective sequential pattern decreases. In the first experiment, task complexity in terms of sequence length and structural complexity was set at a level that was supposedly high enough to induce sleep-related performance enhancement. In the second experiment, sequence length was reduced by 50%. In the third experiment, structural complexity was reduced by organizing the sequence in a much more regular fashion, while sequence length was the same as in Experiment 1. Reducing the task complexity in Experiments 2 and 3 was expected to also reduce the memory load associated with learning these tasks to such an extent that sleep-related offline learning would come into effect only to a lesser degree than in Experiment 1, or maybe even not at all.

## Materials and Methods

All three experiments followed the same experimental paradigm. They were conducted in accordance with the ethical standards of the 1975 Declaration of Helsinki and were approved by the Ethics Committee of Faculty 5 Empirical Social Sciences of Saarland University.

### Participants

A total of 73 young and healthy men and women participated in our studies. Of these, 24 subjects (22.77 ± 2.45 years; 12 men, one left-handed; 12 women) participated in Experiment 1. Another 24 subjects (22.38 ± 2.08 years; 13 men, two left-handed; 11 women, one left-handed) participated in Experiment 2. The remaining 25 subjects (21.64 ± 0.96 years; 16 men, one left-handed; 9 women, two left-handed) participated in Experiment 3. In each experiment, the participants of either sex were randomly assigned to two experimental groups of 12 subjects each in Experiments 1 and 2 and groups of 12 (four female) or 13 (five female) subjects in Experiment 3. Experimental groups were always balanced as far as possible with respect to the participants’ sex.

The subjects had no prior knowledge of the criterion task and were naïve about the hypotheses of the experiments. They were required to refrain from daytime naps, alcohol, excessive caffeine intake and any other drugs from 24 h before their first training session until the end of the last test. Physical activity (e.g., sport practice) was permitted. Their participation was credited as partial fulfillment of the course requirements. There was no additional reward or remuneration. Written informed consent was obtained from all participants prior to their experiment.

### Tasks and Apparatus

**Figure 1:**
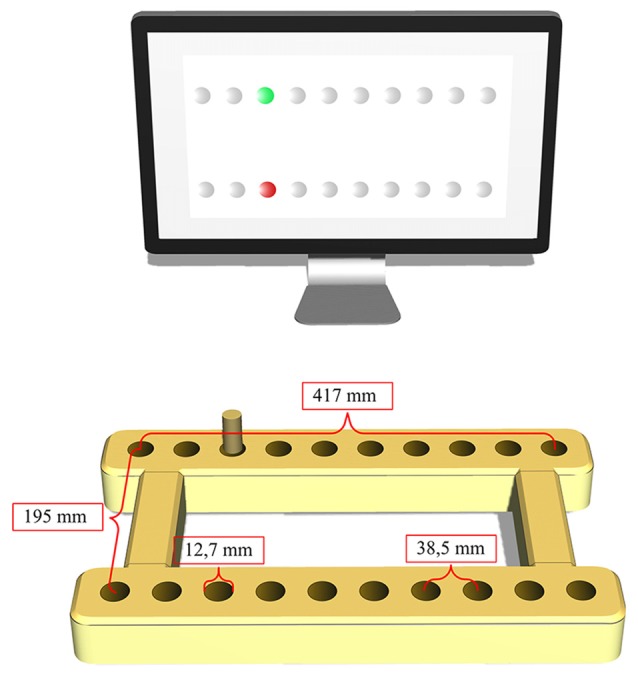
Experimental apparatus (pegboard and monitor). The present peg-location (green) and the new target to be reached for (red) are represented on the monitor.

In each of the experiments, during the task execution, the subjects were seated in front of a table-mounted *electronic pegboard* that was placed horizontally in front of them with their upper trunks strapped to the backrest. Thus, while their body position was fixated with respect to the pegboard, the participants could use their entire arms by freely moving the shoulder, elbow and wrist. The pegboard consisted of two horizontal wooden bars (41.7 cm long, 16 cm apart), each containing 10 holes that were 22.22 mm in depth, 12.7 mm in diameter, 25.4 mm apart in the left-right and 195 mm apart in the forward-backward dimensions (see Figure 1).

The *criterion tasks* employed in each of the experiments required the participants to carry out a spatially defined *arm-movement sequence*. To this end, the subjects were to execute a series of goal-directed movements with a small wooden peg (9.52 mm in diameter and 50.8 mm long) held with a pincer grip in the non-dominant hand. At the end of each reaching movement, they had to quickly fit the peg into a designated hole on the pegboard, thereby closing the magnetic contact. Once the contact was closed, the respective sequence element was terminated, and the next transportation movement had to be started immediately until the sequence was completed. Precision requirements of the reaching movements were determined by calculating their index of difficulty (ID; Fitts, 1954). This was done according to the equation Log_2_(2A/W), where A represents the movement amplitude measured from one target center to the other, and W represents the target width. IDs >4.5 are regarded as high (Boyle and Shea, 2011).

**Figure 2:**
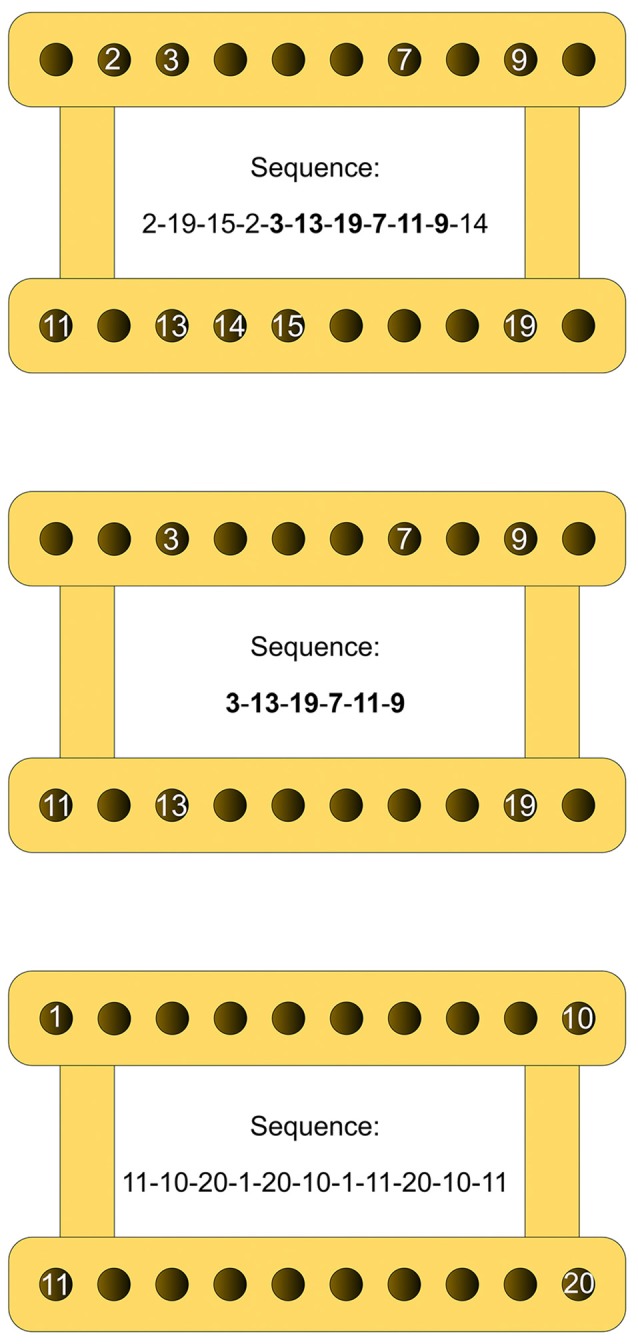
Schematic delineation of the three criterion sequences. Upper panel: sequence employed in Experiment 1; middle panel: sequence employed in Experiment 2; lower panel: sequence employed in Experiment 3. The numbers indicate the spatial locations on the pegboard to be reached for one by one, defining the respective arm movement sequences. Note that figures are presented here only for explanatory purposes; during the experiments, the participants were never presented any numbers.

We used *three different criterion sequences*, one for each experiment (see Figure 2): (a) in *Experiment 1*, the subjects had to carry out a *ten-element* sequence, which followed a spatial pattern *void of any apparent regularity* (Figure 2, upper panel). Reaching movements differed with respect to direction (left, right, forward, backward and diagonal) as well as amplitude (range: 3.83 cm to 36.87 cm). No two movements were identical. Their mean ID amounted to 5.04 (±0.95); (b) in *Experiment 2*, the subjects had to perform a *five-element* sequence (Figure 2, middle panel). The respective movements were identical to elements 5 through 9 of the sequence employed in Experiment 1. They also followed a spatial pattern *without any apparent regularity* and differed in direction and amplitude (range: 20.50 cm to 36.87 cm; mean ID: 5.35 (±0.36)); and (c) in *Experiment 3*, the criterion sequence again included *ten elements* (Figure 2, lower panel). Nine out of 10 reaching movements again differed in direction and amplitude (range: 20.50 cm to 40.10 cm). Elements five and nine were identical. This time, the mean ID was 5.57 (±0.49) and the spatial pattern to be followed included two familiar geometric forms (an x-shape and a rectangle), which made the sequential pattern fairly regular. Additionally, to reduce ambiguity in target identification, each reaching movement terminated at one of the four pegboard corners and nowhere in between.

The participants were explicitly told that a sequence with a fixed number of reaching movements was used consistently throughout the experiment. However, the sequence was never presented entirely before or during execution. Rather, the participants had to learn the task by repeated execution. That is, during acquisition and retention, the targets were visualized one after the other prior to each reaching movement on a computer screen. Correct execution of a sequence element was indicated by a color change of the respective target stimulus from red to green, while the next target symbol was illuminated in red. In case of an error, the symbol representing the target that had been missed turned green as well, while the next target was red. Thus, explicit error control always required participants to compare the peg’s present position on the pegboard to the target position indicated on the screen. Throughout one sequence trial, only the target stimulus of the one element that had just been executed was illuminated in green. Thus, the participants were always presented with stimuli specifying the starting point (green) and end point (red) of just one sequence element at the time. When one sequence was finished, the subjects were to place the peg back into the starting position again (the first position of the sequence indicated by a green symbol on the computer screen) and mentally prepare for the next trial’s execution. After signaling that they were ready, the experimenter gave a verbal start signal about 2 s later. The next trial was then performed until a block of 10 trials had been completed. Sequence configuration, raw data assessment and the screen display during the sequence execution were controlled by means of LMD software (Wagner; IAT Leipzig).

### Design and Procedure

Before initial training, in all three experiments, the subjects were briefly familiarized with the apparatus and the peg-plugging procedure in general. To this end, with their non-dominant hand, they conducted 10 trials of a three-element sequence with elements different from those of the criterion task. Following familiarization, the participants were informed about the length of the respective criterion sequence, and they were told that the sequence was invariant across trials and trial blocks. Thereafter, the participants in each experimental group received initial training in the criterion task (10 blocks of 10 trials each). To prevent fatigue, the trial blocks were always separated by 30-s intervals. The subjects were retested following a 12-h retention interval, with the retest comprising three blocks of 10 trials each. During the retest, the same stimulus information was available to the subjects as during the initial practice. In each experiment, one group of subjects received initial training in the morning (7–9 a.m.) and was retested again in the evening. This group was labeled the ME (= *M*orning-*E*vening) group. The other group practiced in the evening (7–9 p.m.), was retested the next morning, and was labeled the EM (= *E*vening-*M*orning) group correspondingly. Thus, the subjects in the ME groups had to stay awake during a 12-h retention period until the retest (no naps allowed), while those in the EM groups had a regular night’s sleep during their 12-h retention interval. For subjects assigned to the EM groups, the duration and quality of each subject’s sleep during the experimental night was assessed with a standardized sleep questionnaire (Goertelmeyer, 1986). For subjects assigned to the ME groups, daytime activities were assessed with a time-line protocol, which was checked at Retest. The participants were always instructed to perform each movement sequence as quickly and with as few errors as possible. However, they were also advised not to speed up performance at the expense of an increasing error rate. *Performance measures* were the number of *E*rroneous *S*equences (ES) per trial block and *T*otal *E*xecution *T*ime (TET) per sequence, with TET averaged for each subject across the correct sequences in a trial block. The TET is thereby inversely proportional to the sequence execution speed.

### Data Analysis and Statistics

In all three experiments, changes in performance during the acquisition and retention were analyzed with reference to three different time points, comprising three trial blocks each. These were “Start-of-Practice” (blocks 1, 2 and 3), “End-of-Practice” (blocks 8, 9 and 10) and “Retest” (blocks 11, 12 and 13). For each individual and dependent variable, the performance measures were averaged across the respective trial blocks comprised at each time point. The group means were then calculated from the individual subjects’ mean ES and TET per time point during the initial training and retest, respectively. Moreover, to render offline performance changes from different experiments directly comparable, the change scores were calculated for the two retention periods (sleep/wake). To this end, for each participant, the difference in the TET values from “End-of-Practice” to “Retest”, divided by “End-of-Practice”, was calculated. Multiplication by 100 yielded the respective %-values. Normalization of the difference in TET performance across the Time Points (“End-of-Practice”, “Retest”) to the performance achieved by End-of-Practice also adjusts the offline performance differences to the theoretical “room for change” that may differ across individuals.

For inferential statistics, two-way analysis of variance (ANOVA) and paired *t*-tests were run. With respect to repeated-measures factors, in the case of violation of the sphericity assumption, a *df*-correction according to Greenhouse-Geisser was applied. A significance level of *p* < 0.05 was used for all inferential statistics. The effect sizes were provided in terms of $\eta_{\text{p}}^{\text{2}}$ with respect to the ANOVAs and Cohen’s *d* with respect to the *t*-tests. In the case of multiple hypothesis testing using *t*-tests, the *p*-values were Bonferroni-corrected. One-sample *t*-tests were used to compare the change scores against zero. All calculations were conducted with SPSS-PC, version 15.0 (SPSS Inc., Chicago, IL, USA).

## Results

The average sleep duration in the EM groups amounted to 7.00 (±0.51) h in Experiment 1, 6.50 (±0.50) h in Experiment 2 and 6.45 (±0.40) h in Experiment 3. There was no indication of poor sleep quality for any of the participants in any of the experiments. Additionally, no naps or peculiarities were reported for any of the participants assigned to the respective ME groups.

**Figure 3:**
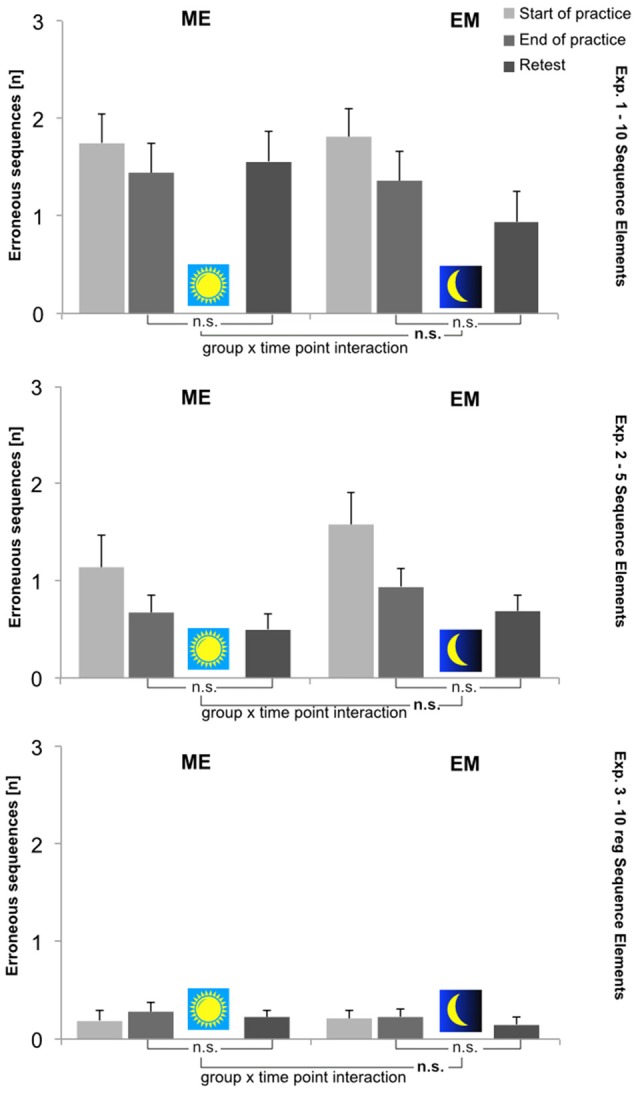
Mean number of erroneous sequences (ES [n]) per group (ME, EM) and time point (Start-of-Practice: block 1, 2 and 3; End-of-Practice: block 8, 9 and 10; Retest (after 12 h): block 11, 12 and 13). Upper panel: Experiment 1; middle panel: Experiment 2; lower panel: Experiment 3. Error bars: standard errors of the mean. Depicted are also statistical results with respect to performance changes during retention (i.e., End-of-Practice to Retest).

In all three experiments, the mean *error score (ES)* in general was low throughout acquisition (Experiment 1: 1.6 (±0.2); Experiment 2: 0.9 (±0.4); and Experiment 3: 0.2 (±0.04)) and remained unchanged throughout the retention intervals. In this respect, experimental groups did not differ, nor was there a Group × Time Point interaction in any of the studies. In all three studies, the error score remained unaffected by the treatment conditions (i.e., wake or sleep during retention). The ES results for all three experiments at each time point are shown in Figure 3.

**Figure 4:**
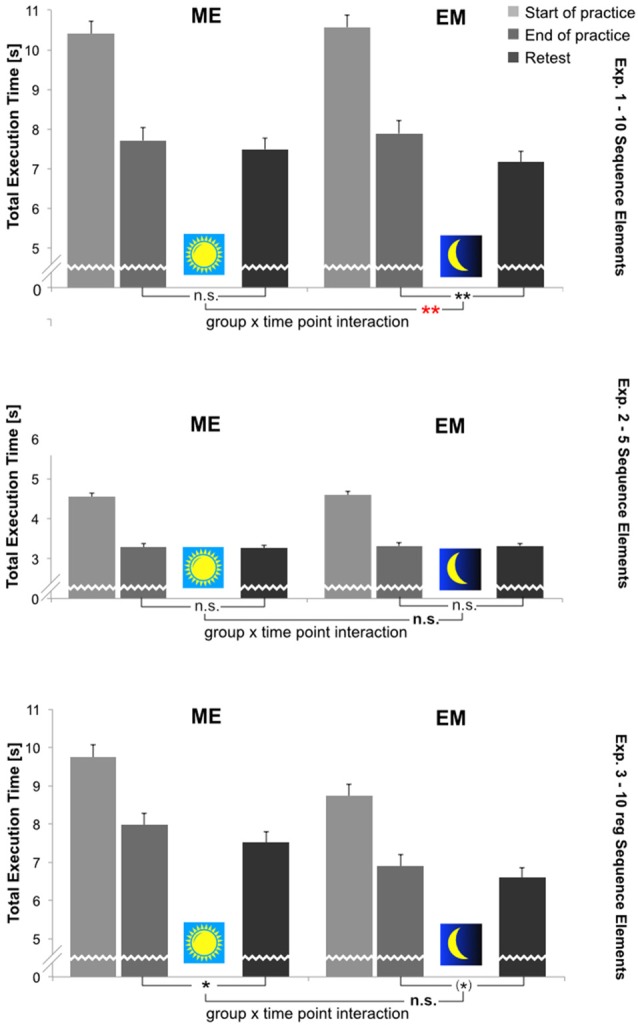
Mean total sequence execution time (TET [s]; correct sequences only) per group (ME, EM) and time point (Start-of-Practice: block 1, 2 and 3; End-of-Practice: block 8, 9 and 10; Retest (after 12 h): block 11, 12 and 13). Upper panel: Experiment 1; middle panel: Experiment 2; lower panel: Experiment 3. Error bars: standard errors of the mean. Depicted are also statistical results with respect to performance changes during retention (i.e., End-of-Practice to Retest). **p < 0.01; *p < 0.05 (Bonferroni-corrected: *p* < 0.025); (*): 0.1 > *p* > 0.05 (Bonferroni-corrected: 0.05 > *p* > 0.025).

*Sequence execution time (TET)*, to the contrary, significantly decreased during acquisition in each experiment. During retention, the TET significantly decreased once more in Experiments 1 and 3. This was not the case in Experiment 2. A significant Group × Time Point interaction for TET changes from the End-of-Practice to Retest was found only in Experiment 1, indicating a significantly greater reduction in TET at Retest in the EM group following sleep than in the ME group after waking. The TET results for all three experiments at each time point are depicted in Figure 4. For a more detailed inspection, for each experiment the time course of TET change across all 10 practice blocks and the three Retest blocks is shown in the Supplementary Figure S1. There was no indication of a speed-accuracy trade-off in any of the experiments. Below, detailed statistical results are reported separately for each experiment.

### Experiment 1

#### Acquisition

For both dependent variables, performance during initial training (i.e., from Start-of-Practice to End-of-Practice) was assessed by 2[Group] × 2[Time Point] ANOVAs with repeated measures on the last factor: (a) the *error score (ES)* was low from the beginning and decreased slightly, though not significantly, from the Start to End of Practice (*p* = 0.128). The groups did not differ (*p* = 0.968), nor was there a Group × Time Point interaction (*p* = 0.773); and (b) in contrast, the *sequence execution time (TET)* significantly decreased during acquisition (*F*_[Time-Point](1,22)_ = 330.44, *p* < 0.001, $\eta_{\text{p}}^{\text{2}}$ = 0.94). However, there was neither a Group × Time Point interaction (*p* = 0.928), nor was TET any different as a function of groups (*p* = 0.686). The TET performance did not level off at the end of acquisition: as became evident from a 2[Group] × 3[Block] ANOVA, the TET significantly decreased even across the last three practice blocks (*F*_[Block](2,44)_ = 12.33, *p* < 0.001, $\eta_{\text{p}}^{\text{2}}$ = 0.36), while neither the Block × Group interaction nor the factor Group reached statistical significance (*p*_[Group]_ = 0.688; *p*_[Block × Group]_ = 0.17). According to the respective pairwise comparisons, the TET significantly decreased from trial block to trial block until the end of practice (*p* ≤ 0.024).

#### Retention

Again, for both dependent variables, performance during retention (i.e., from End-of-Practice to Retest) was assessed by 2[Group] × 2[Time Point] ANOVAs with repeated measures on the last factor. The overall *error score (ES)* did not change further during retention (*p* = 0.447). The groups did not differ (*p* = 0.384), nor was there a significant Group × Time point interaction (*p* = 0.195). Thus, throughout the 12-h retention interval, ES never deviated from its lower asymptote that was reached at the end of practice. *TET* was significantly shorter at Retest compared to End-of-Practice (*F*_(1,22)_ = 28.38, *p* < 0.001, $\eta_{\text{p}}^{\text{2}}$ = 0.56). There was a significant Group × Time Point interaction (*F*_(1,22)_ = 8.27, *p* = 0.009, $\eta_{\text{p}}^{\text{2}}$ = 0.27), while the groups did not differ (*p* = 0.866). That is, the TET reduction at the Retest was significantly greater in the EM group than in the ME group. This result was confirmed by two paired *t*-tests, which were calculated for each group separately to compare the sequence execution time at the End-of-Practice and Retest. Here, a Bonferroni-corrected significance level of *p* < 0.025 was applied. It was shown that TET significantly decreased only during the sleep-filled retention interval in the EM group (*t*_(11)_ = 5.16, *p*_[two-tailed]_ < 0.001, *d* = 1.49) but not during the waking interval in the ME group (*t*_(11)_ = 2.01, *p*_[two-tailed]_ = 0.069, *d* = 0.58). The respective TET-change scores from the End-of-Practice to Retest amounted to 8.9% in the EM group (*p*_[two-tailed]_ < 0.001) and 4.1% in the ME group (*p*_[two-tailed]_ = 0.114).

### Experiment 2

#### Acquisition

For both dependent variables, performance during initial training (i.e., from Start-of-Practice to End-of-Practice) was assessed again by 2[Group] × 2[Time Point] ANOVAs with repeated measures on the last factor. The *error score (ES)* significantly decreased from the Start to End of Practice (*F*_[Time-Point](1,22)_ = 9.41, *p* = 0.006, $\eta_{\text{p}}^{\text{2}}$ = 0.30). The groups did not differ (*p* = 0.280), nor was there a Group × Time Point interaction (*p* = 0.650). The number of ES per trial block dropped rapidly to a value <1 after only 20 practice trials. After having reached that asymptote, the second half of the acquisition period ES remained fairly constant in both experimental groups. This was shown by a 2[Group] × 5[Block] ANOVA calculated for blocks 6–10 (*p*_[Block]_ = 0.533; *p*_[Group]_ = 0.085; *p*_[Block × Group]_ = 0.628). The *sequence execution time (TET)* also decreased significantly during acquisition (*F*_[Time-Point](1,22)_ = 459.92, *p* < 0.001, $\eta_{\text{p}}^{\text{2}}$ = 0.95), while again, neither the factor Group (*p* = 0.724), nor the interaction term (*p* = 0.937) reached statistical significance. Contrary to the error score, during the acquisition the TET never reached a performance asymptote. Instead, even across the last three practice blocks (i.e., blocks 8–10), the TET decreased significantly, as was shown by a 2[Group] × 3[Block] ANOVA (*F*_[Block](2,44)_ = 4.70, *p* = 0.014, $\eta_{\text{p}}^{\text{2}}$ = 0.17; *p*_[Group]_ = 0.756; *p*_[Block × Group]_ = 0.692).

#### Retention

Performance during retention (i.e., from End-of-Practice to Retest) for both dependent variables was assessed by 2[Group] × 2[Time Point] ANOVAs with repeated measures on the last factor. There was no further significant change in the *error score (ES)* during retention (*p* = 0.098), nor was there a significant group difference (*p* = 0.273) or Group × Time Point interaction (*p* = 0.733). Additionally, the *sequence execution time (TET)* did not change throughout the 12-h retention interval but remained virtually constant (*p* = 0.892). Again, there were no group differences (*p* = 0.681) nor a Group × Time Point interaction (*p* = 0.856). The respective TET-change scores from the End-of-Practice to Retest amounted to −0.4% in the EM group and in the ME group (*p*_[two-tailed]_ ≥ 0.77).

### Experiment 3

#### Acquisition

Again, for both dependent variables, performance during initial training (i.e., from Start-of-Practice to End-of-Practice) was assessed by 2[Group] × 2[Time Point] ANOVAs with repeated measures on the last factor. The *error score (ES)* was almost negligible right from the beginning. There were no more than 2.5% ES per trial block throughout acquisition, and the ES did not change significantly from the Start to End of Practice (*p* = 0.380). The groups did not differ (*p* = 0.873), nor was there a Group × Time Point interaction (*p* = 0.640). Contrary to this, the *sequence execution time (TET)* significantly differed as a function of groups (*F*_[Group](1,23)_ = 6.01, *p* = 0.022, $\eta_{\text{p}}^{\text{2}}$ = 0.20) and significantly decreased during acquisition (*F*_[Time-Point](1,23)_ = 197.01, *p* < 0.001, $\eta_{\text{p}}^{\text{2}}$ = 0.89). These performance improvements were about identical in both experimental groups, as was shown by a non-significant Group × Time Point interaction (*p* = 0.924). Other than ES, the TET continuously decreased across the trial blocks and never reached an asymptote. In each experimental group, there was a significant decrease in TET even across the last three practice blocks (i.e., blocks 8–10), as was shown by a 2[Group] × 3[Block] ANOVA (*F*_[Block](2,46)_ = 3.29, *p* = 0.046, $\eta_{\text{p}}^{\text{2}}$ = 0.12; *F*_[Group](1,23)_ = 5.63, *p* = 0.026, $\eta_{\text{p}}^{\text{2}}$ = 0.19; *p*_[Block × Group]_ = 0.488).

#### Retention

Performance during retention (i.e., from End-of-Practice to Retest) for both dependent variables was assessed by 2[Group] × 2[Time Point] ANOVAs with repeated measures on the last factor. There was no further significant change in the *error score (ES)* during retention (*p* = 0.216), nor was there a significant group difference (*p* = 0.580) or a Group × Time Point interaction (*p* = 0.839). However, the *sequence execution time (TET)* significantly decreased again (*F*_(1,23)_ = 14.80, *p* = 0.001, $\eta_{\text{p}}^{\text{2}}$ = 0.39). The groups differed as before (*F*_(1,23)_ = 5.96, *p* = 0.023, $\eta_{\text{p}}^{\text{2}}$ = 0.20), but again, the interaction term failed to reach statistical significance (*p* = 0.500), indicating that TET-reduction throughout the 12-h retention interval was about the same in both experimental groups. The TET-offline improvements in both groups were quite similar and rather small. According to the paired *t*-tests calculated for each group separately, only those in the ME group were significant at a Bonferroni-corrected significance level of *p* < 0.025 (EM group: *t*_(12)_ = 2.52, *p*_[two-tailed]_ = 0.027, *d* = 0.71; ME group: *t*_(11)_ = 2.87, *p*_[two-tailed]_ = 0.015, *d* = 0.82). The respective TET-change scores from the End-of-Practice to Retest amounted to 4.1% (*p*_[two-tailed]_ = 0.025) in the EM group and 4.9% (*p*_[two-tailed]_ = 0.013) in the ME group.

## Discussion

In the motor domain, task complexity is thought to be associated with the control requirements imposed on the human neuromotor system. In the initial stages of motor learning, task complexity is also thought to affect memory consolidation. A specific role in memory consolidation has been attributed to sleep-related processes enhancing the respective skill representation during retention in the absence of any further physical practice. These processes are thought to be causal for marked performance enhancements that have repeatedly been found at Retest only following sleep but not following waking (sleep-related offline learning). The objective of the three studies presented here was to identify whether task complexity affects sleep-related performance improvements in gross motor skills. Two different components of task complexity were addressed, namely, *sequence length* and *structural complexity*, with the latter being a measure of sequence regularity. For gross motor tasks, the sequences of unrestrained arm movements were employed. Each sequence was composed of a certain number of goal-directed reaching movements following a specific spatial pattern. These reaching movements are regarded as the basic units of behavior (i.e., sequence elements). In adults, they are largely pre-programmed before initiation (Gordon et al., 1994) and follow a fixed kinematic pattern (reaching synergy; see Konczak and Dichgans, 1997). During execution, the redundant degrees of freedom then are constrained by subconscious, lower-level control mechanisms to achieve optimal trajectory control and to compensate for any transient perturbations, thereby following a minimum cost principle (Cruse et al., 1993; Dounskaia and Wang, 2014). Initially these reaching movements are separate intentional acts. Organized in a defined ordinal succession they constitute the fundamental pattern of the respective gross motor skill.

The three arm movement sequences were modified according to each experiment’s complexity requirements: (a) in *Experiment 1*, we used a 10-element sequence (nine different target locations) following a non-regular spatial pattern. The same sequence had been used in a previous study (Malangré and Blischke, 2016). In this study, sleep-related offline learning had been found when the participants were retested under free recall conditions. Therefore, sleep-related offline learning was also expected to occur in the present study, with stimulus information still available at retest; (b) in *Experiment 2*, only the *sequence length* was altered by reducing the number of sequence elements from 10 to 5. Although the structural task complexity remained unchanged, this shorter sequence was expected to be much simpler and easier to learn; and (c) in *Experiment 3*, the sequence length was the same as in Experiment 1; only the *structural complexity* was reduced by arranging the sequence elements according to the regular geometric forms. The resulting pattern was expected to be learned and remembered much easier than the one employed in Experiment 1. In reaching movements of this type, movement time depends on their ID (Fitts, 1954). To avoid precision requirements confounding the total sequence execution time (TET), the mean ID was kept about the same in the respective arm movement sequences.

In all three experiments, the participants initially practiced criterion tasks either in the morning (the ME group) or in the evening (the EM group) and were retested 12 h later. Thus, during retention, the EM groups were afforded a night of sleep while the ME groups had to stay awake. The dependent variables were performance error (i.e., number of *ES* per trial block) and *TET* per sequence (correct sequences only). Thus, performance increases as the error score and/or execution time decreases. Note that TET reduction is proportional to an increase in sequence execution speed. The amount of practice and trial number at the Retests were identical in all experiments. In all three experiments, the error score was low from the beginning and remained stable until end of practice without any between-group differences. Across the delay period, there were no significant differences in performance error within or between groups. This indicates that no loss of accuracy accompanied improvements in the sequence execution speed. Instead, all performance differences/changes were exclusively reflected by the TET. The TET significantly decreased during acquisition in all three experiments. At Retest, in Experiment 1, the TET significantly decreased again following sleep but not after waking. Any such offline improvements were completely eliminated, however, when the sequence length was reduced to only five elements in Experiment 2. In Experiment 3, small but significant offline improvements in terms of TET reduction occurred again, but this time, these improvements were of the same magnitude following sleep or waking. Moreover, offline improvements in Experiment 3 were only half the size of those following sleep in Experiment 1.

According to these results, sleep-related offline learning appears to be associated with a sufficient amount of task complexity. In contrast, when the task complexity is sufficiently reduced by either reducing the sequence length or increasing the regularities underlying sequence production, sleep-related performance enhancements are not observed any longer. However, before coming to a final conclusion, several methodological issues first need to be discussed. With respect to motor sequence learning, it has recently been argued that there is little evidence for a performance gain that can be attributed to sleep when confounding variables are factored out that are independent of any possible sleep consolidation effect (e.g., Pan and Rickard, 2015). Here, confounds due to online learning during retests, data averaging and reactive inhibition have been found to be of prominent significance. We will briefly address these aspects in the following section.

In a wide variety of task domains, performance improvement both within and between sessions is known to follow a smooth, monotonically decreasing curve (Newell and Rosenbloom, 1981). Accordingly, Pan and Rickard (2015) have proposed to solve the problem of an *online learning* confound by fitting an appropriate empirical function to each subject’s practice data. The gain score analysis then can be based on the difference between the predicted retest performance (based on the extrapolation of the practice data fit) and the observed performance on the retest blocks. Power law functions (*f*(x) = ax^−α^) have been used previously to estimate test performance in previous studies concerning sleep-related offline learning (e.g., Adi-Japha et al., 2014; Malangré et al., 2014). The assumption of performance improvements closely resembling a power law function holds true also with respect to our own data. This was clearly shown in a study with eight subjects that extensively practiced the movement sequence used in our present Experiment 1 for 600 trials, which was distributed over 3 days (Schmitz and Waßmuth, 2013; unpublished data). The TET continuously decreased following a power function and approached an asymptote of approximately 5.7 s on average only after 550 trials (Supplementary Figure S2). We therefore employed curve fitting to the TET data also in the present case. According to this approach (see Supplementary data for details), sleep-related offline learning appears to be confirmed in Experiment 1. Here, the observed retest performance is significantly superior to the predicted performance in the EM group. Conversely, in the ME group, the respective TET data account for continued online learning following the wake retention interval. That is, only those individuals experiencing a night of sleep revealed improvements in a sequence execution speed at retest beyond what was predicted by individual learning functions derived from their initial practice performance. In Experiment 2, the observed TET-performance at retest was slightly worse than the predicted one. This may be due to a pronounced warm-up decrement evident in both experimental groups in the first retest trial block (see Supplementary Figure S1). In Experiment 3, continued online learning accounts for the slight performance increments that were found at retest. In both of the experimental groups the observed performance measures and the predicted performance measures are about the same.

*Data averaging* procedures are commonly used to assess post-sleep gain throughout the motor consolidation literature because they effectively compensate local performance fluctuations. Therefore, we also averaged performance data at each time point over three trial blocks. However, with respect to testing the sleep-related enhancement hypothesis, data averaging can also profoundly bias results. Averaging over the steeper section of the performance curve early in practice may exacerbate online learning confounds. Moreover, averaging can also be highly sensitive to transient performance patterns, such as warm-up effects on initial test blocks. According to Pan and Rickard (2015), addressing such warm-up effects by just eliminating data from the first test block prior to the calculation of change scores would be ill-advised because it may even exacerbate the online learning confound discussed above. Instead, these authors recommend curve fitting as the one approach that can fully resolve the respective confound due to data averaging.

It is well known that massed practiced conditions can result in an accumulating decline in performance, which is often associated with scalloped *reactive inhibition* effects (for an overview, see Pan and Rickard, 2015). Therefore, any offline gain observed following a delay period at retest may result from the differences in magnitude of reactive inhibition at the end of practice vs. the beginning of the test and may not require a sleep consolidation interpretation. However, reactive inhibition effects can resolve relatively quickly when brief breaks are inserted between blocks of training or when post-training performance is assessed only a few minutes after the end of practice. In our present data, we did not find any evidence of scalloped reactive inhibition. Rather, within practice blocks, early and late trials do not statistically differ, while across practice blocks, performance increases even at the end of practice. This may be due to the small number of trials per block as well as the fact that our participants were instructed to start a new trial within a block only after they had signaled that they were ready to do so. Moreover, in the already mentioned study by Malangré and Blischke (2016) with the same arm movement sequence employed as in our present Experiment 1, post-training measures were assessed under free recall conditions 15 min after practice. After that time, according to the material reviewed by Pan and Rickard (2015), any reactive inhibition effects would have entirely resolved already. Nevertheless, in that study, performance significantly *increased* once more relative to this early post-training test by approximately 10% when the subjects were retested again following sleep and appeared to be stabilized after waking. These results also are in clear support of the sleep-related performance enhancement hypothesis. Thus, all in all, we do not think that reactive inhibition really affected our present data.

There are some other factors that have also been considered to be possible offline learning confounds. According to Pan and Rickard (2015), the *time of training* is not critical to the observed gain score, specifically when the effects of morning vs. evening training times are compared. To fully equate training and testing sessions with respect to physiological circadian and homeostatic factors, however, it appears prudent to use a 24-h delay design. This is what we did in two previous studies using the same task as in Experiment 1 (Malangré and Blischke, 2016) or a task similar to that (Malangré et al., 2014). Independent of time of training, in both of these studies, significant performance improvements were found following sleep but not after waking. We therefore thought it sufficient to limit our present experimental design to a 12-h retention period. Within this time frame, the findings from our present Experiment 1 replicate the previous results well. Conversely, the *time of testing* may have a larger influence on the post-delay gain. For instance, Nettersheim et al. (2015) recently presented data suggesting a transient performance boost in finger-tapping skills 30 min after the end of practice. According to their findings, sleeping immediately afterwards stabilized performance at this level but did not enhance it. However, sleep applied considerably later enhanced performance relative to that assessed at the end of practice only to the “transient boost” level. These and other findings (e.g., Landry et al., 2016) call into question some concepts concerning sleep-related mechanisms of motor memory consolidation that are presently debated. For the time being, however, generalizing these findings across motor task domains would probably be precipitous. In this respect, future work will certainly be needed.

So far, we have dealt with critical issues basically related to the chosen study design. However, another point at issue relates to the *type of motor sequence representation*, which is supposed to be enhanced offline while subjects are asleep. According to the widely accepted model initially proposed by Hikosaka et al. (1999), the acquisition of sequential behaviors resides in the interaction between different neural networks that would encode the same motor sequence in two different coordinate systems (i.e., spatial and motor). One memory component is created rapidly early during training and is thought to incorporate allocentric (spatial) coordinates (e.g., spatial locations of end effectors and/or sequential target positions) and to constitute an abstract effector-independent representation of a series of movements that need to be executed in an external frame of reference. The respective representational code relies on attention, explicit knowledge and working memory. The other memory component develops more slowly and is supposed to be mediated through egocentric (motor) coordinates (e.g., sequence of activation patterns of the agonist/antagonist muscles and/or achieved joint angles) and thus should constitute an effector-dependent, movement-based skill realized in an internal frame of reference. This representation relies on implicit knowledge and does not require attention or working memory. Hikosaka’s model has also been applied to research motor memory consolidation. There, it has been shown that *sleep* specifically favors enhancement of the *extrinsic* (spatial) sequence representation, while consolidation of the respective *intrinsic* (motor) representation was *not* modulated by the sleep/wake condition (Witt et al., 2010; Albouy et al., 2013). According to this model, and considering that after only 100 sequence execution trials our subjects still had only little practice, we conjecture performance improvements observed in our experiments to be mainly due to enhancement of the abstract spatial sequence representation and its declarative knowledge base.

Still another caveat might be raised considering our chosen main criterion variable, namely, *TET*. As long as participants have not yet fully memorized the spatial sequence pattern specifying the target locations on the pegboard, they need to visually register each new target stimulus on the computer screen and then find its counterpart on the pegboard. Thus, it could be argued that our participants’ performance improvements (i.e., the TET-reduction) substantially resulted from improving their gaze behavior. However, in our opinion this does not call into question the validity of TET (i.e., sequence execution time) as a global performance measure. The visual target location is always an inherent component of any reaching movement executed in Euclidean space. The respective saccades and reaching movements are usually initiated simultaneously, with the saccades being terminated earlier than the limb movement (Jeannerod, 1997). The visual target location must therefore be regarded an integral part of the sequence execution skill at any stage of expertise. Early on, however, the subjects are still in the process of acquiring a cognitive, abstract spatial sequence representation (Hikosaka et al., 1999). During that stage, the gaze moves back and forth between the computer screen and work space, and a visual search will necessarily add to TET as the overall performance variable. However, once a sequence pattern has been learned, subjects do not need to rely on visual stimulus information any longer. This has been demonstrated in the previously mentioned study by Malangré and Blischke (2016), where sleep-related offline improvements have been shown under free recall conditions.

In that previous study, the criterion task was either identical to (Experiment 1) or more difficult than the tasks used in our present study (Experiments 2 and 3). At the same time, however, the study by Malangré and Blischke (2016) involved 20% more practice trials (i.e., a total of 120 trials) than our present experiments, and even then, six out of a total of 24 participants eventually had to be excluded from the final analysis for not having fully memorized the sequence. We therefore assume that in our present study, at least in Experiment 1, a considerable albeit unknown number of participants had not yet fully learned the sequence prior to Retest. These subjects are likely to have relied on visual checks and cuing again when being retested. The time attributed to this gaze behavior therefore could have obscured the true effect of sleep-related memory consolidation to some extent. Moreover, suchlike incomplete declarative learning during acquisition in all probability was more pronounced in Experiment 1 than in the two other experiments, because their tasks were easier and could be memorized much sooner. We therefore have to acknowledge that different levels of explicit sequence knowledge in the different experiments might also have contributed to the differential sleep-dependent improvement. These limitations call for a careful interpretation of our present results, and certainly more research is needed in order to corroborate our present findings.

All this considered, we still believe that our present data provide some new evidence on the relationship between task complexity and motor memory consolidation. More specifically, for the first time, we have shown that the number of elements and structural complexity *independently* affect sleep-related offline performance improvements in a sequential motor task under cued recall conditions. That is, reducing structural complexity in a ten-element sequence indeed had the same effect as reducing the sequence length from 10 to 5 elements, with structural complexity remaining invariably high. In both cases, sleep-related performance enhancements that are evident in a sufficiently complex arm movement sequence were not observed any longer. Thus, by dissociating different complexity components and by incorporating gross motor tasks, we successfully extended earlier work by Kuriyama et al. (2004).

The question now arises as to how our observations might be interpreted in light of theoretical considerations regarding processes of sleep-related motor memory consolidation. One possible answer might be found in relation to processing demands associated with sequence acquisition, the participants’ working memory capacity, and subsequent processes of sleep-related memory enhancement. Sequences that are easy to remember might not profit as much from active system consolidation processes as sequences that clearly exceed working memory capacity. Sequences of up to five independent elements (as in Experiment 2) are well within adult subjects’ memory span (Mathy and Feldman, 2012) and thus are easy to remember during acquisition. This might have limited the performance-enhancing effect of subsequent consolidation processes. On the other hand, while longer sequences are more difficult to remember, the simplicity of patterns within a sequence wields an independent influence: a sequence of given length is more difficult to remember as the number of distinct subsets (chunks) increases. Conversely, fewer and larger subsets within a sequence reduce its structural complexity (and increase its formal “compressibility”; see Mathy and Feldman, 2012). Accordingly, the complexity of a sequence expresses how much memory space is required to encode it and determines the success of memory processes. Again, if a sequence representation is already well-established during initial practice due to its low structural complexity, subsequent consolidation processes may not be able to induce any further performance improvements. Specifically, with respect to Experiment 3, we conjectured that tracing familiar geometric forms in the course of sequence execution would promote the use of cognitive strategies, which in turn could serve as binding rules to integrate the individual task components into a meaningful “gestalt”. The respective geometric arrangement of a set of separate reaching movements would then immediately be conceptualized as a unified representation (Franz et al., 2001; Swinnen and Wenderoth, 2004) and effectively reduce the structural task complexity. As a consequence, we believe that a sufficiently stable sequence representation was already established during practice and that subsequent memory consolidation affected the performance at retest to a lesser degree. Of course, all these considerations are only preliminary at present and need to be carefully scrutinized in the course of future research.

## Study Limitations

There are also some limitations to our study: (a) one is the lack of polysomnographic data. In future studies, it would be interesting to see the extent to which the impact of motor task complexity on offline learning in a gross motor task is also systematically reflected in polysomnographic measures such as, for example, sleep-spindle density; (b) another limitation arises from the fact that we did not assess our participants’ individual working memory capacity. In the context of the above considerations, it appears reasonable to expect task complexity and working memory capacity to interact in their impact on motor memory consolidation at the individual level as well; (c) we also did not systematically assess our participants’ cognitive retrieval strategies. This could have contributed to a better understanding of the declarative knowledge underlying successful sequence retrieval; (d) furthermore, we experimentally addressed only two components of motor task complexity so far (i.e., the number of sequence elements and structural complexity). Incorporating components such as, for example, the number of end-effectors or inter-limb coordination, was beyond the scope of our present study. However, these aspects are specifically relevant to tasks in applied areas such as sports and vocational training, stroke rehabilitation and physical therapy; and (e) last, in future studies, assessment of gaze behavior and kinematic data could elucidate how different components involved in gross motor sequence execution might contribute differently to performance improvements due to practice and subsequent memory consolidation.

## Author Contributions

Both authors contributed extensively and equally to the work presented in this manuscript; discussed and commented on the manuscript at all stages. KB and AM developed the research topic and designed the experiments; prepared, analyzed data and discussed results. AM supervised data collection. KB wrote the article.

## Conflict of Interest Statement

The authors declare that the research was conducted in the absence of any commercial or financial relationships that could be construed as a potential conflict of interest.

## References

[B1] Adi-Japha E. Badir R. Dorfberger S. Karni A. (2014). A matter of time: rapid motor memory stabilization in childhood. Dev. Sci. 17, 424–433. 10.1111/desc.12132 24620995

[B2] Albouy G. Fogel S. King B. R. Laventure S. Benali H. Karni A.. (2015). Maintaining vs. enhancing motor sequence memories: respective roles of striatal and hippocampal systems. Neuroimage 108, 423–434. 10.1016/j.neuroimage.2014.12.049 25542533

[B3] Albouy G. Fogel S. Pottiez H. Nguyen V. A. Ray L. Lungu O.. (2013). Daytime sleep enhances consolidation of the spatial but not motoric representation of motor sequence memory. PLoS One 8:e52805. 10.1371/journal.pone.0052805 23300993 PMC3534707

[B4] Al-Sharman A. Siengsukon C. F. (2013). Sleep enhances learning of a functional motor task in young adults. Phys. Ther. 93, 1625–1635. 10.2522/ptj.20120502 23907080

[B5] Backhaus W. Braaß H. Renné T. Krüger C. Gerloff C. Hummel F. C. (2016). Daytime sleep has no effect on the time course of motor sequence and visuomotor adaptation learning. Neurobiol. Learn. Mem. 131, 147–154. 10.1016/j.nlm.2016.03.017 27021017

[B6] Born J. Wilhelm I. (2012). System consolidation of memory during sleep. Psychol. Res. 76, 192–203. 10.1007/s00426-011-0335-6 21541757 PMC3278619

[B7] Boyle J. B. Shea C. H. (2011). Wrist and arm movements of varying difficulties. Acta Psychol. 137, 382–396. 10.1016/j.actpsy.2011.04.008 21600531

[B8] Cohen D. A. Pascual-Leone A. Press D. Z. Robertson E. M. (2005). Off-line learning of motor skill memory: a double dissociation of goal and movement. Proc. Natl. Acad. Sci. U S A 102, 18237–18241. 10.1073/pnas.0506072102 16330773 PMC1312380

[B9] Cruse H. Brüewer M. Dean J. (1993). Control of three- and four-joint arm movement: strategies for a manipulator with redundant degrees of freedom. J. Mot. Behav. 25, 131–139. 10.1080/00222895.1993.9942044 12581984

[B10] Dounskaia N. Wang W. (2014). A preferred pattern of joint coordination during arm movements with redundant degrees of freedom. J. Neurophyiol. 112, 1040–1053. 10.1152/jn.00082.2014 24872537

[B11] Doyon J. Korman M. Morin A. Dostie V. Tahar A. Benali H.. (2009). Contribution of night and day sleep vs. simple passage of time to the consolidation of motor sequence and visuomotor adaptation learning. Exp. Brain Res. 195, 15–26. 10.1007/s00221-009-1748-y 19277618 PMC2752878

[B12] Fischer S. Nitschke M. F. Melchert U. H. Erdmann C. Born J. (2005). Motor memory consolidation in sleep shapes more effective neural representations. J. Neurosci. 25, 11248–11255. 10.1523/JNEUROSCI.1743-05.2005 16339020 PMC6725908

[B13] Fitts P. M. (1954). The information capacity of the human motor system in controlling the amplitude of movement. J. Exp. Psychol. 47, 381–391. 10.1037/h0055392 13174710

[B14] Franz E. A. Zelaznik H. N. Swinnen S. S. Walters C. (2001). Spatial conceptual influences on the coordination of bimanual actions: when a dual task becomes a single task. J. Mot. Behav. 33, 103–112. 10.1080/00222890109601906 11265060

[B15] Genzel L. Kroes M. C. W. Dresler M. Battaglia F. P. (2014). Light sleep versus slow wave sleep in memory consolidation: a question of global versus local processes? Trends Neurosci. 37, 10–19. 10.1016/j.tins.2013.10.002 24210928

[B16] Genzel L. Quack A. Jäger E. Konrad B. Steiger A. Dresler M. (2012). Complex motor sequence skills profit from sleep. Neuropsychobiology 66, 237–243. 10.1159/000341878 23095374

[B17] Goertelmeyer R. (1986). “Schlaf-Fragebogen A und B (Sf-A, Sf-B),” in Collegium Internationale Psychiatriae Scalarum, 3rd Edn. ed. International Skalen für Psychiatrie (Weinheim: Beltz).

[B18] Gordon J. Ghilardi M. F. Ghez C. (1994). Accuracy of planar reaching movements: I. Independence of direction and extent variability. Exp. Brain Res. 99, 97–111. 10.1007/bf00241415 7925800

[B19] Gudberg C. Wulff K. Johansen-Berg H. (2015). Sleep-related motor memory consolidation in older adults depends on task demands. Neurobiol. Aging 36, 1409–1416. 10.1016/j.neurobiolaging.2014.12.014 25618616 PMC4353561

[B20] Hikosaka O. Nakahara K. Rand M. K. Sakai K. Lu X. Nakamura K.. (1999). Parallel neural networks for learning sequential procedures. Trends Neurosci. 22, 464–471. 10.1016/s0166-2236(99)01439-3 10481194

[B21] Hoedlmoser K. Birklbauer J. Schabus M. Eibenberger P. Rigler S. Mueller E. (2015). The impact of diurnal sleep on the consolidation of a complex gross motor adaptation task. J. Sleep Res. 24, 100–109. 10.1111/jsr.12207 25256866 PMC4491357

[B22] Jeannerod M. (1997). The Cognitive Neuroscience of Action. Cambridge, MA: Blackwell.

[B23] Kempler L. Richmond J. L. (2012). Effect of sleep on gross motor memory. Memory 20, 907–914. 10.1080/09658211.2012.711837 22901032

[B24] Konczak J. Dichgans J. (1997). The development towards stereotype arm kinematics during reaching in the first three years of life. Exp. Brain Res. 117, 346–354. 10.1007/s002210050228 9419079

[B25] Kuriyama K. Stickgold R. Walker M. P. (2004). Sleep-dependent learning and motor-skill complexity. Learn. Mem. 11, 705–713. 10.1101/lm.76304 15576888 PMC534699

[B26] Landry S. Anderson C. Conduit R. (2016). The effects of sleep, wake activity and time-on-task on offline motor sequence learning. Neurobiol. Learn. Mem. 127, 56–63. 10.1016/j.nlm.2015.11.009 26655281

[B27] Malangré A. Blischke K. (2016). Sleep-related offline improvements in gross motor task performance occur under free recall requirements. Front. Hum. Neurosci. 10:134. 10.3389/fnhum.2016.00134 27065834 PMC4809884

[B28] Malangré A. Leinen P. Blischke K. (2014). Sleep-related offline learning in a complex arm movement sequence. J. Hum. Kinet. 40, 7–20. 10.2478/hukin-2014-0002 25031668 PMC4096088

[B29] Mathy F. Feldman J. (2012). What’s magic about magic numbers? Chunking and data compression in short-term memory. Cognition 122, 346–362. 10.1016/j.cognition.2011.11.003 22176752

[B30] Morita Y. Ogawa K. Uchida S. (2012). The effect of a daytime 2-hour nap on complex motor skill learning. Sleep Biol. Rhythms 10, 302–309. 10.1111/j.1479-8425.2012.00576.x

[B31] Nemeth D. Janacsek K. Londe Z. Ullman M. T. Howard D. V. Howard J. H. Jr. (2010). Sleep has no critical role in implicit motor sequence learning in young and old adults. Exp. Brain Res. 201, 351–358. 10.1007/s00221-009-2024-x 19795111

[B32] Nettersheim A. Hallschmidt M. Born J. Diekelmann S. (2015). The role of sleep in motor sequence consolidation: stabilization rather than enhancement. J. Neurosci. 35, 6696–6702. 10.1523/JNEUROSCI.1236-14.2015 25926448 PMC4412892

[B33] Newell A. Rosenbloom P. S. (1981). “Mechanisms of skill acquisition and the law of practice,” in Cognitive Skills and Their Acquisition, ed. Anderson J. R. (Hillsdale, NJ: Erlbaum), 1–55.

[B34] Pan S. C. Rickard T. C. (2015). Sleep and motor learning: is there room for consolidation? Psychol. Bull. 141, 812–834. 10.1037/bul0000009 25822130

[B35] Robertson E. M. Pascual-Leone A. Press D. Z. (2004). Awareness modifies the skill-learning benefits of sleep. Curr. Biol. 14, 208–212. 10.1016/s0960-9822(04)00039-9 14761652

[B36] Schmitz L. Waßmuth N. (2013). Zum Einfluß der Übungsrate auf Ausführungsgeschwindigkeit und Reproduktionssicherheit einer komplexen Armbewegungsfolge—Eine Laborstudie. Unpublished Bachelors’ Thesis; Department of Sport Science, Saarland University, Germany.

[B37] Smith C. (1995). Sleep states and memory processes. Behav. Brain Res. 69, 137–145. 10.1016/0166-4328(95)00024-n 7546305

[B38] Song S. Howard J. H. Jr. Howard D. V. (2007). Sleep does not benefit probabilistic motor sequence learning. J. Neurosci. 27, 12475–12483. 10.1523/JNEUROSCI.2062-07.2007 18003825 PMC6673329

[B39] Stickgold R. Hobson J. A. Fosse R. Fosse M. (2001). Sleep, learning, and dreams: off-line memory reprocessing. Science 294, 1052–1057. 10.1126/science.1063530 11691983

[B40] Swinnen S. P. Wenderoth N. (2004). Two hands, one brain: cognitive neuroscience of bimanual skill. Trends Cogn. Sci. 8, 18–25. 10.1016/j.tics.2003.10.017 14697399

[B41] Walker M. P. (2005). A refined model of sleep and the time course of memory formation. Behav. Brain Sci. 28, 51–64; discussion 64–104. 10.1017/s0140525x05000026 16047457

[B42] Walker M. P. Brakefield T. Morgan A. Hobson J. Stickgold R. (2002). Practice with sleep makes perfect: sleep-dependent motor skill learning. Neuron 35, 205–211. 10.1016/S0896-6273(02)00746-8 12123620

[B43] Witt K. Margraf N. Bieber C. Born J. Deuschl G. (2010). Sleep consolidates the effector-independent representation of a motor skill. Neuroscience 171, 227–234. 10.1016/j.neuroscience.2010.07.062 20691764

