# Supplementary material for: Task Complexity Modulates Sleep-Related Offline Learning in Sequential Motor Skills
Source: Front Hum Neurosci. 2017 Jul 25;11:374. doi: 10.3389/fnhum.2017.00374 (PMC5525265; doi:10.3389/fnhum.2017.00374)
Supplement: Supplementary file 1 [file Presentation_1.pdf]

Supplemental Material

to

Blischke, K. and Malangré, A.

Task complexity modulates sleep-related offline learning in sequential motor skills

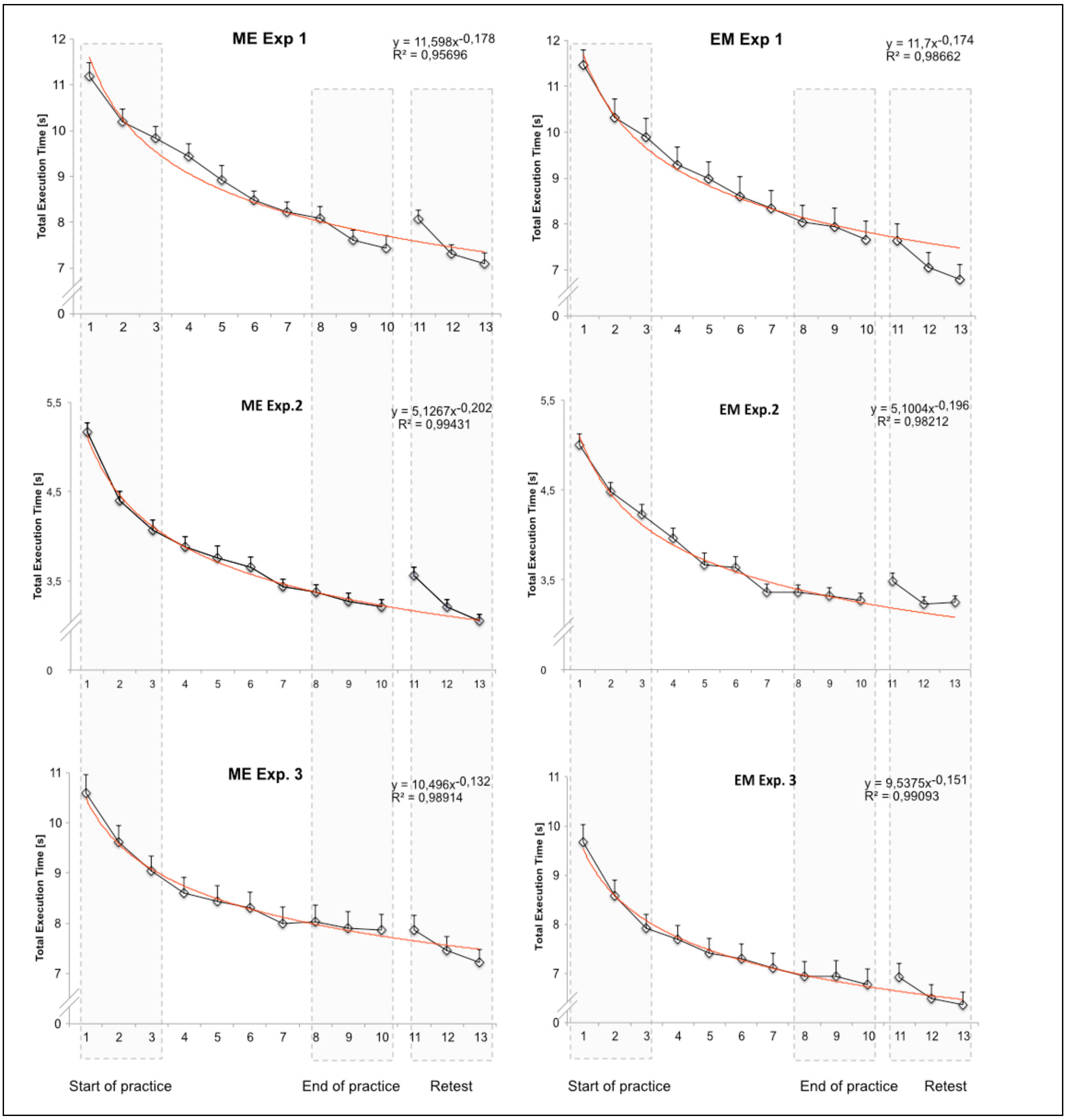

Supplemental Figure 1: Mean Total Execution Time (TET; seconds) per experiment (experiment 1: upper panel; experiment 2: middle panel; experiment 3: lower panel), groups (ME-groups: left; EM-groups: right) and trial block (acquisition: blocks 1 through 10; retention: blocks 11 through 13; correct sequences only). Symbols represent group means per trial block (observed performance); error bars: standard errors of the mean. Solid red lines represent power functions derived from participants' acquisition data. Their extensions are used to predict performance resembling continued learning during the additional three trial blocks at retention. Shaded areas depict time points (Start-of-Practice: block 1, 2 & 3; End-of-Practice: block 8, 9 & 10; Retest: block 11, 12 & 13.

Total Execution Time (TET):
Comparison of observed and predicted Retest-measures

In the present study, we wanted to determine, whether any significant reductions in participants' TET (i.e. improvement in execution speed) at retest(s) in our experiments was a result of any offline enhancement consolidation, or just a consequence of further practice. One way to assess enhancement consolidation considering continued learning at retention, involves extrapolation of each subject's respective initial training data by means of power law functions. Power law functions can be used to mathematically model practice-dependent changes in performance in the course of skill acquisition (Newell and Rosenbloom, 1981). By extrapolation of this practice data fit participants' performance at retest(s) can be estimated as if practice had just continued without any pause and under the same conditions that were present during training. Suchlike *predicted* retention data can then be used in conjunction with the individuals' *observed* performance on the retest trials. If the observed performance is better (i.e. TET lower) than the predicted performance, offline facilitation is assumed to have occurred.

In the present study, *predicted* TET-Retest-measures were provided as follows: based on each single subject's TET-acquisition data (means per trial block), for each individual a power function of the type $y = kn^{-c}$ was calculated and used to obtain an estimate for that individual's performance for the additional three trial blocks during Retest. Here k is the mean value of TET on the first practice trial block, c is the learning rate (with $c < 0$), and n is the number of trial blocks. Predicted TET-data for each individual then were collapsed across blocks, thus providing mean predicted TET-performance at Retest for each one subject. Thus, if sleep (but not wake) had indeed enhanced memory consolidation, *observed* TET should turn out significantly *shorter* as compared to predicted TET in the EM-groups when tested right after the sleep-filled 12 hrs retention interval, but not in the ME-groups when tested following the 12 hrs wake retention interval. Special consideration has also been given to the possibility that the first Retest block (i.e. block11) might yield performance data, which are systematically confounded by a warm-up decrement. The respective results for each of the three experiments are as follows:

Experiment 1: According to a 2[Group] x 2[Data-Type] ANOVAs with repeated measures on the factor "Data-Type" (TET-observed, TET-predicted), calculated on each group's observed and predicted TET-data at Retest, the observed TET-data in experiment 1 turned out shorter than the predicted ones. This difference nearly reached statistical significance (F (1, 22) = 4.20, p = .052, η^2_p = .16). While groups did not differ (p = .818), the Group x Data-Type interaction was significant (F (1, 22) = 5.48, p = .029, η^2_p = .19). As can be inferred from Table 1, this significant interaction effect can be attributed to the observed TET being shorter than the predicted TET only in the EM-group, while in the ME-group observed and predicted TET-values apparently did not differ. This finding was corroborated again by paired t-tests calculated for each group separately in order to compare observed and predicted TET-data at Retest. With a Bonferroni-corrected significance level of $p < .025$, these t-tests turned out significant only in the EM-group (t (11) = -2.94, $p_{[two-tailed]}$ = .013, d = .85), but not in the ME-group (t (11) = .21, $p_{[two-tailed]}$ = .831). The same results were achieved for each group when these calculations were conducted without trial block 11 (i.e. the first Retest block), and performance measures at Retest for each participant were averaged only across trial blocks 12 and 13 in order to control for a possible warm-up decrement (EM-group (t (11) = -3.64, $p_{[two-tailed]}$ = .004, d = 1.44; ME-group (t (11) = -1.58, $p_{[two-tailed]}$ = .141).

Experiment 2: Again, observed and predicted TET-Retest-measures were compared by means of a 2[Group] x 2[Data-Type] ANOVAs with repeated measures on the factor Data-Type (TET-observed, TET-predicted). Contrary to any á-priori expectations, observed sequence execution time in both experimental groups of experiment 2 proved to be slightly, but significantly *longer* at Retest than the predicted one. This was shown by a significant factor Data-Type (F (1, 22) = 19.977, $p < .001$, η^2_p = .476), while neither the factor Group (p = .756), nor the interaction term (p = .723) did reach statistical significance. The surprising result of observed TET-data being *longer* at Retest than the predicted ones was also corroborated by paired t-tests calculated for each group separately, comparing observed and predicted TET-data at Retest. With a Bonferroni-corrected significance level of $p < .025$, these t-tests turned out significant in the EM-group (t (11) = 2.82, $p_{[two-tailed]}$ = .017, d = .81) as well as in the ME-group (t (11) = 3.99, $p_{[two-tailed]}$ = .002, d = 1.13). As is apparent from the descriptive data presented in Supplemental Figure 1, there is a pronounced warm-up

decrement present in the first of the three trial blocks at Retest (i.e. in block 11) in both groups of experiment 2. This may have caused the average observed TET-data at Retest being slightly longer than the predicted ones.

To control for this possible warm-up effect, observed and predicted TET-data at Retest were compared once more for each group separately without trial block 11. To this end for each participant the respective performance measures were averaged only across trial blocks 12 and 13. According to the respective *t*-tests, in both groups observed and predicted TET-data at Retest statistically did not differ any more (EM-group (t (11) = 2.04, $p_{[two\text{-}tailed]}$ = .067; ME-group (t (11) = 1.17, $p_{[two\text{-}tailed]}$ = .265). Thus, it seems that participants have compensated the warm-up decrement evident in block 11 by achieving TET-values in block 12 and in block 13 that about matched the predicted ones.

Experiment 3: Again, based on individual power law functions derived from each participant's acquisition data, *predicted* TET-values were calculated as an estimate of each individual's performance at Retest, if just continued learning had taken place. Observed and predicted TET-Retest-measures then were compared by means of a 2[Group] x 2[Data-Type] ANOVAs with repeated measures on the factor "Data-Type" (TET-observed, TET-predicted). As a result, in each experimental group of experiment 3 observed and predicted sequence execution time at Retest were actually the same ($p_{[Data\text{-}Type]}$ = .897; $p_{[Group\ x\ Data\text{-}Type]}$ = .618), while groups as such again significantly differed ($F_{[Group]}$ (1, 23) = 5.8545, p = .024, η^2_p = .203). The central finding of observed and predicted TET-data at Retest being about the same, was corroborated again by paired *t*-tests calculated for each group separately ($p_{[two\text{-}tailed]} = \geq .671$). The same results were also achieved for each group when these calculations were conducted without trial block 11 (i.e. the first Retest block) in order to control for a possible warm-up decrement ($p_{[two\text{-}tailed]} = \geq .217$).

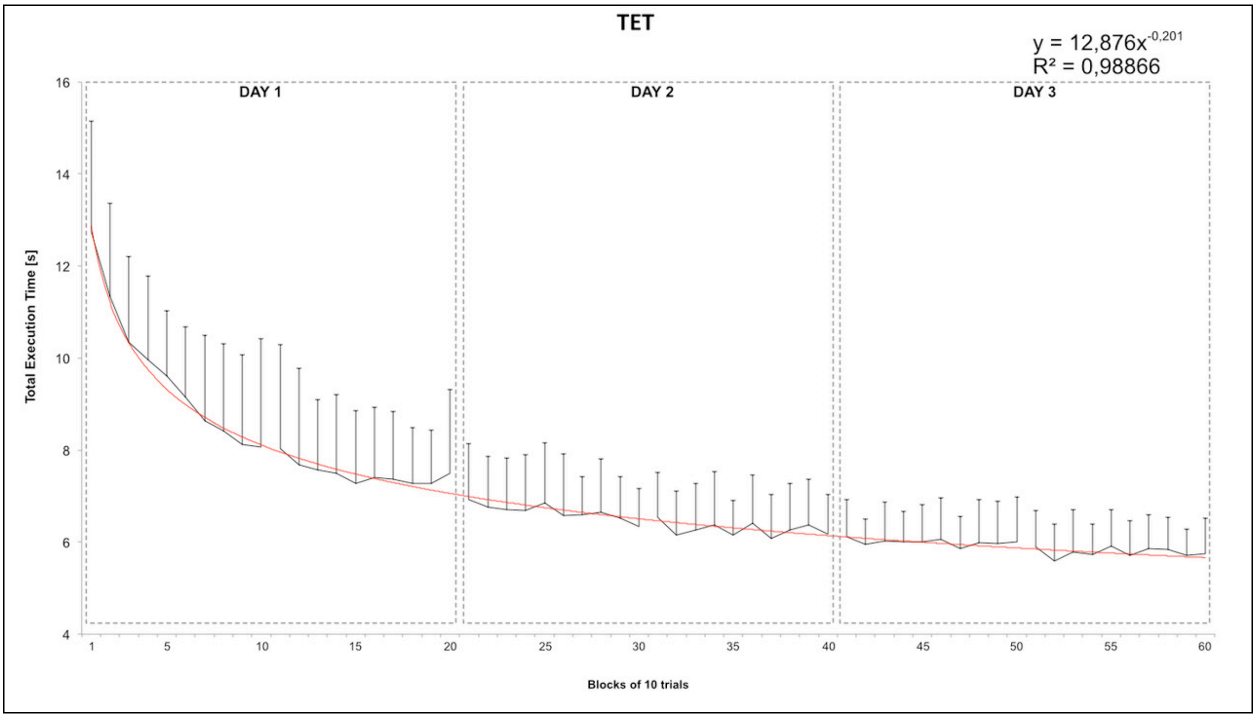

Supplemental Figure 2: Eight young, healthy participants (four males, four females; age: $\bar{x}$ = 23.13 years, range: 19 to 26 years) extensively practiced the ten-element arm movement sequence used also in experiment 1. They executed 600 trials, distributed in blocks of ten trials over three consecutive days. Trial blocks were separated by a 30 sec pause. Each day comprised two practice sessions of 100 trials each, separated by a half-hour break. Stimulus information was always provided. Presented are the means of total sequence execution time (TET [s]; correct sequences only) per block of ten trials, averaged across trials and participants. Error bars are standard deviations. The red curve resembles a power function fitted through the trial block mean values. Note that any deterioration of performance possibly due to fatigue or loss of motivation is apparent only after 180 trials at the end of the second practice session on day one. (Adapted from Schmitz, L., and Waßmuth, N. (2013). *Zum Einfluß der Übungsrate auf Ausführungsgeschwindigkeit und Reproduktions-sicherheit einer komplexen Armbewegungsfolge – Eine Laborstudie*. Unpublished Bachelors' Thesis; Saarland University, Department of Sport Science.)
